# Military Sexual Trauma: Gender, Military Cultures, and the Medicalization of Abuse in Contemporary America

**DOI:** 10.1080/17526272.2021.1884785

**Published:** 2021-02-22

**Authors:** Joanna Bourke

**Affiliations:** Birkbeck, University of London, UK

**Keywords:** sexual violence, medicalization, US military, trauma, gender, rank, military law

## Abstract

Sexual violence is a serious problem within armed services. This article explores intra-service rape in branches of the U.S. military from the 1990s to the present. The article begins by establishing the parameters of the crisis of sexual abuse within the U.S. armed services. Second, it explores systematic failures to recognize forms of suffering. Victim-survivors in the military are vulnerable to military-specific obstacles to reporting their abuse and being believed. Attention is paid to differences by gender and sexual orientation. Third, it analyses the medicalization of suffering in the modern military and its effects. What meanings are assigned to ‘military sexual trauma' (MST) and how has that label affected victim-survivors of rape or sexual assault? The article concludes by arguing that the concept of ‘trauma’ as it is applied to victims of sexual abuse does a formidable amount of political and ideological work.

‘I am a survivor of military sexual trauma’, began the anonymous author of an article on abuse in the U.S. armed forces. Her abuser, she continued, ‘outranked me by several grades and had been in the military for years’. She had signed a contract with the military that did not allow her to simply quit the job, so he became ‘a constant figure in my life for the next few years’. The sexual assaults took place while she was in uniform and in her workplace. Not only did she repeatedly tell her abuser to stop, she also told colleagues about what he was doing. They simply informed her that they knew that he was abusing other servicewomen as well; they did not want to get involved. She was told, ‘Look, everyone knows he does this stuff. Do you want to ruin your career and OUR career because you have an issue with it?’ Her abuser warned that he would ‘rate me poorly on evaluation [and] counseling statements to indicate that I was trouble’ and he threatened to ‘create rumors that I was having sex with enlisted soldiers or with my married male friends’. She finally discovered that there were services within the military where she could seek help but, when she attempted to contact them, the telephone number did not work and the person responsible for providing assistance had been transferred (‘Being my Own Virgil’ 2015: 339–41).

The testimony of this anonymous author alludes to some arguments I will be making in this article, including the denial and dismissal of sexual violence in the U.S. military and the pathologization of victims. The article focuses on *intra*-service rape – that is, rape carried out by servicepersonnel against other servicepersonnel. It begins by establishing the parameters of the crisis of sexual abuse within the U.S. armed services. Second, it explores systematic failures to recognize forms of suffering. Third, it analyses the medicalization of suffering in the modern military and its effects.

The terms used to write about sexual harms are complicated. I will be using concepts like rape, sexual violence, sexual assault, and sexual abuse interchangeably. This is only shorthand. It is not intended to imply that these acts are identical. But it is equally not to assume that there is a continuum in seriousness, starting with emotional hurt and ending with sadistic violence. This article is also not concerned with the hierarchies of harms that pervade legal texts and public discussions (for example, notions that physical wounds are ‘worse’ than psychological harms or that women can be ‘raped’ while men are ‘assaulted’). Rather, I seek to draw attention to the unique nature of suffering for each victim-survivor for whom the physical and psychological are enmeshed. I also use concepts like ‘victim’ and ‘survivor’ cautiously. After all, not all victims are survivors: psychological trauma belongs to those who are not killed. However, the emphasis on victimhood can end up defining individuals solely in terms of the actions of another person, trapping them forever in the ‘bad event’. As a result, the traumatized person risks being defined by ‘it’, the ‘bad event’. The term ‘victim-survivor’ is useful, therefore, to draw attention to the fact that people who experience harms are much more than the sum of their vulnerabilities.

## Denial and dismissal

Military cultures are suffused with sexual violence. The earliest feminist texts in the field – those of Germaine Greer, Kate Millett, and Susan Griffin, for example – identified masculinity as a category of analysis, criticizing people gendered male for their insouciant cruelty and casual sense of sexual entitlement (Greer, 1970; Griffin, 1971; Millett, 1971). But it wasn’t until 1975, when Susan Brownmiller published her classic *Against Our Will*, that the *distinctive* risks associated with war and military cultures were highlighted (Brownmiller, 1975). Since then, military contexts have generated a vast and sophisticated literature in the disciplines of history, military studies, international relations, politics, sociology, anthropology, and gender studies. The international research network SVAC (Sexual Violence in Armed Conflicts) is evidence of the degree of interest in the field.

This article focusses on the U.S. military. This is not to deny high levels of sexual violence both within other militaries as well as in non-military contexts. One in five civilian women in the UK and Europe will experience sexual violence in their lifetime (Bourke, 2007). Within armed services, sexual abuse is an even larger problem globally (Morris, 1996). In 2018, for example, the British Army reported that nearly three-quarters of military women had been subjected to inappropriate and unwelcome comments; one-fifth had experienced inappropriate sexual touching; 8 per cent had been involved in a serious sexual assault; and 3 per cent reported being raped. Only 10 per cent of these women made a formal complaint. Even worse: of those complaining 70 per cent were dissatisfied with the outcome (Dodd, 2019). Similarly, in France and Germany, levels of sexual abuse and harassment have skyrocketed, although the extent of the problem has been hampered by the fact that evidence is not systematically collected, let alone reported (Lichfield, 2014; Minano & Pascual, 2014). The global nature of such abuses is explored in a monograph by the author that will be published in November 2021 (Bourke, in press, 2021).

The U.S. is no exception. All studies reveal high levels of sexual abuse in the U.S. military. Official statistics (which are certainly underestimates) released by the Department of Veteran Affairs in 1995 found that six per cent of servicewomen and one per cent of servicemen had experienced an attempted or completed sexual assault (*Healing the Wounds* 2010: 2). When the Department explored the proportion of personnel reporting sexual assault who were using a Veterans’ Affairs (VA) health care service, these levels rose dramatically. A survey of 828 female veterans seeking care at the Baltimore Veterans Affairs Medical Center in the mid-1990s discovered that 41 per cent reported having been raped, 55 per cent had been sexually abused, and 27 per cent reported having been raped, sexually abused, *and* physically abused (Coyle & Wolan, 1996: 588–93). During a congressional hearing in 2010, congress men and women were informed that 23 per cent of female veterans using VA health services had been sexually assaulted (*Healing the Wounds* 2010: 2). Many of these women had been assaulted ‘in the field’. A shocking 15 per cent of servicewomen in Iraq and Afghanistan reported having been sexually assaulted or harassed (*Healing the Wounds* 2010: 2). When another study showed that rates of sexual assault were higher during wartime than they were in peacetimes, a Committee of Veterans’ Affairs concluded that the ‘stress of war’ was responsible (*Healing the Wounds* 2010: 2). In the words of one victim-survivor,

One of the problems over in Iraq for female soldiers is that there is a lot of sexual harassment and rape is huge. And it does not matter if you’re 18 or 58. Women serving over there don’t have to worry about enemy fire. They have to be worried about the guy that’s next to them, you know, that’s supposed to be protecting and taking care of them and a lot of times he becomes like public enemy number one for them (Mattocks et al., 2012: 540).

In the U.S. today, levels of sexual assault are highest in the U.S. Marines and Navy and lowest in the airforce; 18 per cent of completed investigations involved male victims; and 66 per cent of victims were in the lowest rank (US Department of Defense’s Sexual Assault Prevention and Response, 2019: 30. For potential explanations for such differences, see Brown, 2012).

Although sexual violence is clearly not solely a problem for the military, it is particularly widespread in that context. Men who are attracted to armed cultures may be more aggressive than many of their peers. There are also aspects of military culture that foster abuse. Aggression is rewarded; cultures of dominance, routine (Harway & Steel, 2015: 376). There is a vast literature analysing the ways military training regimes facilitate sexual violence, especially of racialized ‘Others’ (Welland, 2013: 881–902). Martial masculinity is inherently sexualized in aggressive ways. This is strongly argued in Aaron Belkin’s classics study *Bring Me Men* (2012), where he exposes a cultural of sexual violence and the hypermasculine endurance of extreme pain, combined with the need to perform a hypermasculine endurance of extreme pain within the U.S. military. This literature also acknowledges that military cultures also share ideologies and norms within the wider society (Sasson-Levy, 2011: 73–98; Szitanyi, 2020). There is good evidence, for example, that many recruits to the U.S. forces had sexually assaulted people *prior* to enlisting. The extent of such pre-military violence was revealed in some research carried out between 1994 and 1997 by researchers at the Naval Health Research Centre. They surveyed 7,850 U.S. navy recruits at the Navy Recruit Training Commands in Orlando (Florida) and Great Lakes (Illinois) and discovered that between 10 and 12 per cent of the recruits admitted committing penile rape prior to entering the navy. A further two to four per cent reported *attempting* to rape a girl or woman. Upon enlisting, these recruits simply continued their abusive practices (Merrill et al., 2001: 252–61).

The sense of crisis around such revelations was intensified by the fact that, from the conflicts in Iraq, Kuwait, and Afghanistan onwards, women were serving in the same units, battalions, and platoons as servicemen. This represented a dramatic change. While at the end of the 1990s, less than five per cent of servicepersonnel were female (‘Testimony Before the Subcommittee on Health’ [hereafter *Government Accountability*] 1998: 1), by 2009, this had risen to 14 per cent and a large proportion were being deployed to combat zones (*Healing the Wounds* 2010: 54). The military was increasingly reliant on the labour of women: public knowledge of the high risk of sexual assault could seriously damage recruitment.

Crucially, though, the crisis was not only about risks to servicewomen. The abuse of men in the armed services continues to be ignored, minimized, or denied. In an analysis of 74 articles discussing sexual abuse in the U.S. military and published in the period up to 2009, only two focused exclusively on the sexual harms done to male servicepersonnel (Allard et al., 2011: 324–45).

Many feminist scholars argue that there are good pragmatic reasons to be wary about focusing on male victims (Bourke 2007, 2021). After all, systems of justice (and even more so *military* justice) are already deeply skewed against women who accuse men of sexual abuse. A hardheaded feminist politics might not wish to tip the balance against female victims even more than it already is. However, a feminist politics that turns a blind eye to the vulnerability of the full range of bodies, of all genders, is diminished as a result.

In the case of the military, ignoring male victims is particularly egregious. No one doubts that servicewomen are at *much* higher risk of being sexually harmed. However, in 2010, 46 per cent of all veterans diagnosed with ‘military sexual trauma’ (MST) were men as were nearly 40 per cent of those being treated for MST (Department of Veterans Affairs, Office of Mental Health Services, 2011). In 2013, evidence was given before the House of Representatives that 54 per cent of the 26,000 victims of sexual assaults while on active duty were men (Committee on Veterans’ Affairs, *Safety for Survivors* [hereafter *Safety for Survivors*] 2013: 8). There is also considerable evidence that military men suffer *higher* levels of personal impairment after sexual attack than do female servicemembers (O’Brien et al., 2015: 358).

The relative absence of abused men (as opposed to women) in the literature is partly because they face particular barriers to having their abuse acknowledged. What is ‘sexual abuse’? The number of sexually abused men dramatically increases if the definition of ‘sexual’ goes beyond forced penile penetration to include coerced genital activity or abusive contact with sexual organs more generally. For instance, sexualized violence against men (including being hit in the testicles) is a common component of training regimes.

The labels are given to the sexual abuse of men also serve to mask harms. Within the U.S. army, navy, and airforce, for example, acts of ‘hazing’ are common. They are called ‘rites of passage’ rather than ‘sexual violence’, despite the fact that they may involve coerced masturbation, the beating of the genitals or buttocks, being required to imitate or perform fellatio, and the forcible insertion of objects inside anuses (Bourke, 2016: 56–64). If those abuses were perpetrated against women, there would be no question that they would be labelled ‘sexually abusive’. Even worse: in U.S. forces, hazing is publicly *defended* on the grounds that it is essential in forging the combat-ready soldier.

## Low reportage

A large proportion of victims of military sexual assault *never* report being attacked to their superiors. Estimates of non-reportage soar as high as 90 per cent, making it a more serious problem than it is in civilian contexts (*Healing the Wounds*, 2010, 1 and 7). According to the Department of Defense’s Sexual Assault Prevention and Response report for 2018, 37 per cent of female servicemembers who were estimated to experience sexual assault reported the crime; the percentage of men was only 17 per cent (U.S. Department of Defense’s Sexual Assault Prevention and Response, 2019: 11).

Even worse, the chairman of a 2012 congressional committee found that three-quarters of servicewomen who *did* report their abuse stated that ‘they would not have made the same decision about reporting the incident again due to the consequences it had on their military career’ (*Invisible Wounds*, 2013: 1). Sixty-two per cent of servicewomen who filed unrestricted reports of assault believed that they *had* suffered retaliation (Wolff & Mills, 2016: 841). According to the Department of Defense’s Sexual Assault Prevention and Response report for 2018, of those victims reporting retaliation as a result of their abuse, 66 per cent claimed the retaliation came from the ‘superior in their chain of command’ (US Department of Defense’s Sexual Assault Prevention and Response, 2019: 41).

Victim-survivors in the armed forces have to counter the full range of harmful myths about sexual violence. Many of these distortions are *not* unique to military cultures but circulate throughout American society. The most important is the belief that ‘women lie’. The view that women are prone to falsely accuse men of rape pervades public and private accounts of abuse. Like victim-survivors elsewhere, military victims are also susceptible to shame and embarrassment.

But, victim-survivors in the military are vulnerable to *additional*, military-specific obstacles to reporting their abuse and being believed. These can be summarized under five general headings: contractual restrictions and ‘chains of command’, organizational cohesion, warrior comportment, interpersonal vulnerability, and gendered and sexual identities.

The most basic difference between victim-survivors of sexual abuse in the military and those in other workplaces (except prisons) is that military personnel usually do not have the option of leaving and there are exacting ‘chains of command’. This is the first explanation for low levels of reporting. Victims do not have recourse to civilian systems of justice but have to rely on military justice. It is often unclear to whom a victim should report to. For many victims (including the anonymous woman with whom I started this article), the first (and only) port of call was the chaplaincy services. But military chaplains do not provide counselling; they simply listen and proffer reassurance.

In other cases, the people to whom a victim is required to report abuse are their senior officers. In other words, the people adjudicating over their fate are senior military figures, most of whom are not trained in the law and have little experience in judging (Belkin, 2012). These officers tend to focus on ‘resistance’ rather than ‘consent’. Furthermore, in one-quarter of cases, the person to who victim-survivors are required to report their abuse is actually the perpetrator (*Invisible Wounds*, 2013: 62). It is common for victim-survivors to be required to continue living and working closely with the person/s who abused them. In the rare cases where perpetrators are identified, they receive slight punishments, often simply being ‘confined to barracks’ (Committee on Veterans’ Affairs, 2013: 5). As one victim-survivor complained, since ‘the barracks he was confined to was the one where I worked’, this constitutes ‘chain of command harassment’ (Committee on Veterans’ Affairs, 2013: 5). In some cases, they were given *Honorable* Discharges (US Department of Defense’s Sexual Assault Prevention and Response, 2019: 27).

Furthermore, it is common knowledge that reporting abuse will harm careers: security clearances can be revoked and the likelihood of being dismissed from the service (with, as argued below, a stigmatizing mental health label) dramatically increases (*Healing the Wounds*, 2010: 42). Victim-survivors in the navy, for example, complained that reporting an assault would ‘set them back in their training schedules or delay them from reporting to their next duty station’ (*NAVINSGEN. Naval Inspector General Report to VCNO* [hereafter NAVINSGEN (2004)] 2004: 21).

For victims there is a high risk of ‘collateral’ damage. After all, many assaults take place in contexts involving the violation of the Uniform Code of Military Justice: victims were drinking, for example, or fraternizing with other ranks. As a result, victims may be subject to further punishment themselves (NAVINSGEN, 2004, i and 39). They are often given ‘Other Than Honorable’ (OTH) separations (or discharges) from the services for reporting their abuse or the abuse of others. Indeed, some accept OTH separations so they will not be required to continue working alongside the perpetrator and his friends (Karin, 2016: 167–68).

Chains of command do not only affect victim-survivors but also those senior personnel responsible for enforcing a ‘zero tolerance’ policy to sexual abuse in their units. *Exposing* abusive behaviour is seen as an indictment of an officer’s leadership qualities and can be used against them in promotion applications (*Healing the Wounds*, 2010, 9 and 14; U.S. Department of Defense, Sexual Assault Prevention and Response Office, 2012). According to one Navy report,

leaders at all levels of the chain of command expressed frustration with what they characterize as a ‘zero tolerance’ mentality for command reporting SA [sexual assault], because they perceive that any reported misconduct reflects negatively on command leadership (i.e. too many SA cases would be analogous to running a ship aground, causing the ultimate relief of the Commanding Officer) (NAVINSGEN, 2004: 30).

Second, the military is an organizational culture like no other. Servicepersonnel are socialized to be exquisitely sensitive to rank and to show immediate obedience to commands from more senior officers. One study found that 80 per cent of the identified assailants possessed a higher rank than their victims (Wolff & Mills, 2016: 844). Was it any wonder that ‘snitching’ is taboo?

Preserving ‘organizational cohesion’ is a key value (*Healing the Wounds*, 2010, 2). In the words of one women who served in the National Guard in Iraq between 2005 and 206, ‘When you’re in a team environment, [and] you report something bad that happened to you, you’re the one responsible for breaking up the team’ (Burns et al., 2014: 347). This poses *particular* problems for women enlisting in an armed service such as the Marines or a combat unit that has a very long and virulent history of hostility to their presence. Complaining about sexual assault is not only deemed to weaken unit cohesion (a classic case of blaming victims) but is also used in arguments about female unsuitability for the job. As one senior warrant office bluntly informed a survivor of military rape, ‘she was the reason why women should not be allowed in the military’ (*Invisible Wounds*, 2013: 24). In other words, the ‘problem’ was women, not failures of discipline within the ranks.

This means that the likelihood of a perpetrator of abuse being punished is abysmally small, especially when a unit is posted to remote locations. ‘What goes on deployment, stays on deployment’, is a pervasive mantra (NAVINSGEN 2004: 26). In the words of a navy report,

There appears to be a type of ‘loyalty code system’ operating within commands, whereby it is tacitly understood that members owe their loyalty to the Navy, the command, and to their superiors. ‘Whistle-blowing’, or reporting unethical and/or criminal acts, effectively labels that person as an outsider, who is not part of the team and cannot be privy to insider information. Repercussions for the whistle blower are not unexpected. According to this code, it is permissible for males to commit criminal acts against females, as the ‘loyalty code’ will protect them and the command image (NAVINSGEN 2004: 34).

The unspoken message is that women are not really part of the team: the loyalty code is male-only. This ‘us versus them’ mentality makes breaching the code of *esprit de corps* particularly treacherous. As victim-survivor Victoria Saunders (who had been raped in 1975 while on active duty in Fort Carson, Colorado) told the 2013 Committee on Veterans’ Affairs,

When you report a rape[,] you become public enemy number one. No one will talk to you. And if they do it is to tell you, you got what you deserved. You are called names, you internalize what happened, and it feels like it is your fault (Committee on Veterans’ Affairs, 2013: 5).

In a workplace which valorizes group cohesion, loyalty, and stoicism, ostracism is a powerful inhibitor of speaking out against fellow ‘brother-in-arms’.

The third reason servicemembers are reluctant to report their abuse is the formidable claim of the warrior code. Both male and female victims believe that reporting victimization makes them ‘weak and less of a warrior’ (*Healing the Wounds*, 2010: 42). Male victims are made to feel that they are less than ‘real men’ (NAVINSGEN, 2004: 21). In the worlds of a poster at the Infantry Battle School, ‘pain is weakness leaving the body’ (Hennessy, 2010: 98). ‘Warriors’ are not only *capable* of ‘sucking it up’, they are publicly *required* to do so in all circumstances.

The fourth explanation emerges from this need to comport oneself as a warrior. When in training or active service, victims are located in environments where everyone is armed, hyper-fit, and trained to fight (including in face-to-face combat). Retribution from perpetrators, their friends, or supporters is not something to dismiss lightly.

The final reason for low levels of reporting is sexual identity. This affects men in the military to an even greater degree than women. According to one study, 65 per cent of service men who had been sexually assaulted developed symptoms of PTSD compared to 39 per cent of men who experienced trauma associated with combat (Kessler et al., 1995: 1048–60. Also see Romaniuk & Loue, 2017: 80–104). Male victims had more symptoms of PTSD than female victims and their symptoms persisted for longer periods (Gaher et al., 2016: 55–62). Indeed, one of the explanations for the fact that abused service*men* reported *higher* levels of personal impairment than their female counterparts is because abused servicemen have to deal with additional issues of masculinity and sexual orientation (that is, the idea that men who are raped are homosexual) (Elder et al., 2017b: 59–66 and Juan et al., 2017: 243–50). Of course, pervasive homophobia in American society means that *civilian* men who are sexually abused also struggle with issues of sexual identity. However, the heightened masculinity and heteronormativity in the military raises these problems to a much higher level. Rape myths (such as the one that men don’t get sexually assaulted) makes it even more shocking and stigmatizing for male victims (O’Brien et al., 2015: 358). A study of Navy men in 2004 revealed that they did not think it was necessary to be taught about sexual assault because ‘things like that do not happen in an all-male crew’ (NAVINSGEN, 2004: 14). In the words of one male navy officer,

If a person assaults another, with no witnesses, and no physical evidence, or in my case I even had physical evidence (punch marks/bruises) it is not worth the repercussions and stigma of our current legal systems to even try to obtain justice. It is better to tell no one and try to forget it (NAVINSGEN, 2004: 35).

Male victim-survivors have also imbued the belief that men ‘initiate and control sexual activities’ (O’Brien et al., 2015: 358). The culture of fitness and strength, which is fostered in the military, makes it difficult for servicemen to accept that they were not strong or aggressive enough to prevent the attack. After all, servicemen are trained to fight. In the words of one male victim-survivor,

I felt guilty about not fighting back. I blamed myself because of that. I mean, he was a big guy and the only thing he said was, ‘Don’t do anything or I’ll cut you up’. I blame myself for not raising hell or fighting or something (Elder et al., 2017a: 202).

They are expected to be tough, unemotional, and willing to suffer for the sake of unit cohesion. The fact that, during the sexual assault, many male victim-survivors had erections and even ejaculated (something that often happens as a result of prostate stimulation) also leads to confusion, dismay, and anxiety (O’Brien et al., 2015: 360). As one male Vietnam veteran who suffered from rectal bleeding for days after being forcibly sodomized confessed, he felt intense shame because he ‘bled like a woman’ (O’Brien et al., 2015: 362).

High levels of shame meant that male victim-survivors are even more likely than their female counterparts to delay seeking treatment and, when in treatment, often conceal the reasons for their trauma (Elder et al., 2017b: 60). Indeed, one study revealed that servicemen in treatment for Military Sexual Trauma often told others that their trauma was combat-related (O’Brien et al., 2015: 360).

Instead of seeking treatment, male veterans often responded to their abuse by a heightened enactment of masculine heterosexuality. In one set of interviews with male victims, published in 2017, the researchers found that, after the assault, the men increasingly engaged in high-risk sexual practices with multiple female partners in order to demonstrate to themselves that they were heterosexual, in control, and hyper-masculine (Elder et al., 2017a: 198–207). In the words of one of their interviewees,

I felt like I had to prove myself, like maybe I was gay and had something written on me I couldn’t see. I remember one time I picked up in one night, three prostitutes in a row and it proved to myself that I was not gay and that I was really attracted to women. I had to … keep proving how macho I was because deep down I thought I was not because of what happened (Elder et al., 2017a: 201).

Their sample also engaged in extravagant masculine behaviours, such as boasting about ‘conquests’, becoming compulsive weight-lifters in order to build muscle, acquiring large tattoos, walking with an exaggerated gait, and refusing to admit to emotions (Elder et al., 2017a: 201–2). Their refusal to talk about what they were feeling impeded ‘the emotional processing critical for treatment for PTSD’ (Elder et al., 2017a: 199), resulting in higher levels of distress than female victims.

Sexualities also matter. Until repealed in 2011, the policy of ‘Don’t Ask, Don’t Tell’ left gay, trans, and queer servicepersonnel especially vulnerable (*Healing the Wounds*, 2010: 15). In the first study of transgender servicepersonnel and mental health in the U.S. military (carried out only in 2016), the researchers found that more than one in five transgender men and one in seven transgender women experienced military sexual trauma (Lindsay et al., 2016: 565). This is certainly an under-estimate given the stigma attached to being transgender in the military and their fears about being denied hormone treatment should they disclose having been abused (Lindsay et al., 2016: 566).

There are numerous barriers placed in the way of trans and other queer people in the military from reporting sexual abuse. The most important one is that disclosing one’s sexual identity would lead to dismissal. Even suspicion of a non cis-gender orientation will provoke discriminatory practices, including being subjected to transphobic insults and insinuations that they are so unattractive that sexual abuse is unlikely (Calton et al., 2016). Unfortunately, transphobic views can be internalized by trans-people, especially within the military where gender identities are policed.

As elsewhere in the world, trans-victims in the U.S. military face special difficulties having their abuse acknowledged and responded to appropriately. Senior officers routinely blame trans-victims for their own abuse. The most notorious example has been dubbed the ‘trans panic defence’ – that is, *perpetrators* of sexual abuse absolve themselves of responsibility on the grounds that they had been deceived into believing that they were engaging in a sexual liaison with a cis-gendered person. The view that trans-people provoke violence by not revealing their birth gender was defended by Bradford Bigler in an article published in the respected *UCLA Law Review* in 2006. He contended that the ‘nature of the sex to which the deceived party consents (for example, heterosexual sodomy)’ was ‘fundamentally different than the act in which the defendant actually engaged (here, homosexual sodomy)’. This means that the *victim* is guilty of fraud; the *aggressor* had simply panicked (Bigler, 2006: 800–1). By such logic, transgender people are always defined by their anatomical sex as decided at birth, a denial both of their sense of self and their lived experience.

## Official responses

These explanations for not reporting sexual assault are exacerbated by the fact that, at every stage in seeking justice, victim-survivors face formidable barriers. Senior officers, not trained lawyers, are designated the arbiters of scientific knowledge. Victim-survivors have to fight against regulations that they can only access medical care for their physical or psychological injuries if they have served for more than two years (*Hearing on War-Related Illnesses* [hereafter *Hearing on War-Related Illnesses]*, 1998: 3). This rule ignores the fact that most abuse takes place in the first few months of training. It also means that Reservists (many of whom had seen active service in war zones) are unable to access help since they are, by definition, unlikely to serve for such a length of time.

When victim-survivors seek treatment for the psychological aftermaths of sexual abuse, they are met with ‘completely unrealistic’ demands of proof. The evidence they are expected to provide to veterans’ committees is significantly more rigorous than what other PTSD sufferers are asked for (where, for example, lay testimony is accepted) (*Invisible Wounds*, 2013: 5). These demands are often impossible anyway since the various branches of the armed services routinely destroy all evidence and investigative records after a short period of time (*Invisible Wounds*, 2013: 5 and 12).

The medical treatment victim-survivors receive is perfunctory: some even call it ‘horribly disrespectful’ (*Safety for Survivors*, 2013: 5, 10–11, 16, and 61). Many states (such as California) have legislation that made it mandatory for health care practitioners to inform the courts of any patient who the practitioner knows or suspects has been a victim of sexual crimes (*Healing the Wounds*, 2010: 44). However, VA medical services are exempted. Medical personnel who are supposed to give victim-survivors advice and practical assistance often prove unsympathetic. This is partly because of role conflicts for Commanders, padres, social workers, and physicians in the military: in other words, responsibility for the care of victims of sexual assault is in conflict with the need to maintain unit cohesion. Inevitably, the force preservation and national interests override other principles, such as the dignity of servicewomen. Military and medical ethics are in conflict.

Even the specially designated clinics for sexually abused veterans are staffed by counsellors who have been trained by the military rather than at civilian rape crisis centres (*Healing the Wounds*, 2010: 6). They are immersed in a ‘military culture that habitually blames the victim and is too often concerned with protecting the image of a platoon or commander’ (*Healing the Wounds*, 2010: 6). According to Helen Benedict, who interviewed forty female veterans in the course of writing *The Lonely Soldier*, sexual assault response coordinators

often treated them [victims] with such suspicion that they felt retraumatized and intimidated out of pursuing justice. Indeed, the usual approach to a report of sexual assault … is to investigate the victim, not the perpetrator and to dismiss the case altogether if alcohol is involved (*Healing the Wounds*, 2010: 6).

In the words of one survivor, ‘it is as if we are the perpetrator’ (*Safety for Survivors*, 2013: 6).

Getting the military authorities to acknowledge the trauma caused by sexual assault is an uphill battle. This is partly because rape trauma is considered trivial compared with combat-induced trauma (*Healing the Wounds*, 2010: 15). As Stephanie Szitanyi argues in her *Gender Trouble in the U.S. Military* (2020), the U.S. military draws a strong distinction between ‘real war wounds’ and marginalized ‘other’ wounds, such as those caused by sexual trauma (2020: chapter 4). This explains why a ‘Freedom of Information’ request found that, between 2008 and 2010, only one third of PTSD claims based on sexual assault were approved, compared with 54 per cent of *other* PTSD claims (*Invisible Wounds*, 2013: 5).

There is also a culture of minimizing the harm of sexual assault, especially when the victims are women. Of those veterans who were lucky enough to have their claims for sexual abuse-induced PTSD accepted, women were likely to receive between 10 and 30 per cent disability ratings while men received 70–100 per cent ratings (*Invisible Wounds*, 2013: 5). The huge gender gap exposes the belief by the Veterans’ Affairs that the sexual abuse of servicemen is more damaging than that of servicewomen.

Having a diagnosis accepted does not guarantee treatment. Veteran’s Affairs have sought to blame rape-trauma on sexual assaults that personnel might have experienced *prior to* entering the services (*Invisible Wounds*, 2013: 28). After all, it is widely known that survivors of sexual assault in the military had high levels of sexual assault *before* joining the military (*Hearing on War-Related Illnesses*, 1998: 25 and 29). A 1998 survey found that one-quarter of women in the U.S. army reported an attempted or completed rape during childhood, a level which is twice as great as amongst the female population more widely (Martin et al., 1998: 213–16). In another study of female veterans, published in 2006, female veterans were much more likely to report having been sexually assaulted by a parental figure before signing up than civilian women. They also experienced longer duration of child sexual abuse. Indeed, 92 per cent of female veterans who had been sexually abused identified a parental figure as the perpetrator compared with only 10 per cent of a community sample (Schultz et al., 2006: 726). Male veterans, too, had twice the odds of experiencing forced sex before the age of 18 compared to men without military service (Blosnich et al., 2014: 1041–48. Also see Wolf-Clark et al., 2017: 121–28).

Some of the reforms intended to increase levels of reporting have backfired. Since 2005, the military has allowed for two forms of reporting: Restricted and Unrestricted. In the former, victims can report their abuse and receive medical care and advocacy services but there would not be an investigation. In contrast, if a report of sexual assault was ‘Unrestricted’, an investigation is triggered. One quarter of all reports in 2014 were restricted ones (Wolff & Mills, 2016: 841). Giving victim-survivors these options did have its ‘desired effect’: the number of people making Restricted Reports increased. However, it shielded the military from having to investigate and punish offenders (Wolff & Mills, 2016: 841). It also did nothing to diminish the focus on victims and their behaviour. Why did victims act in particular ways? Why did they accept a drink? Why didn’t they scream? Any action that accusers failed to take is seen as bestowing responsibility on them for their own abuse. Every part of their agency is scrutinized. In contrast, the accused are rendered passive, merely a prop in a ‘bad story’.

Indeed, the military fails to acknowledge responsibility until exceptionally abusive actions are leaked to the public. Thus, Veterans’ Affairs only began offering sexual trauma counselling in 1993, in the aftermath of the Tailhook scandal in which U.S. naval officers sexually abused 14 female officers and 12 civilians (‘Military Sexual Trauma Counseling Act 1998: 1). The programme was expanded in 1998 – but only after the violence at the Aberdeen Proving Ground was revealed (‘Military Sexual Trauma Counseling Act 1998: 1). Progress has been painfully slow. In 2010, less than ten per cent of all VA medical centers offered inpatient mental health treatment to veterans who had been sexually assaulted (*Healing the Wounds*, 2010: 13). Furthermore, in most cases, treatment takes place in mixed gender groups. In 2010, only 9 out of 153 medical centers offered residential treatment facilities specifically for women suffering from mental health injuries due to sexual trauma (*Healing the Wounds*, 2010: 13). Even then, female victim-survivors found themselves harassed within the medical centres (*Healing the Wounds*, 2010: 14). Patients were not given separate sleeping areas from the male patients (*Healing the Wounds*, 2010: 57). One critic even claimed that victims were examined on a gynaecological table facing towards the examination room door (*Healing the Wounds*, 2010: 57).

Male victims express additional grievances. In 2013, 14,000 of the 26,000 victims of sexual assaults while on active duty were men, but only one of the twelve residential treatment programmes for the treatment of military sexual trauma accepted male patients and, in that case, men were treated alongside women (*Safety for Survivors*, 2013: 8). Outpatient treatment similarly takes place in gender-mixed groups. Male victims were dismayed to discover that responsibility for military sexual trauma programmes was vested in the Director of *Women’s* Mental Health, Family Services, and Military Sexual Trauma (*Safety for Survivors*, 2013: 8). Survivor Brian Lewis complained that this reinforced the message that the Veterans Health Administration regarded rape as a ‘woman’s issue’ (*Safety for Survivors*, 2013: 8). He argued that male as well as female survivors of sexual assault ‘deserve a space to be safe and to not to [*sic*] be triggered’ (*Safety for Survivors*, 2013: 14). In his words, ‘I deserve to have, in essence, my manhood respected by not having to seek my care in a woman’s clinic’ (*Safety for Survivors*, 2013: 14).

## Pathologization

Criticisms about inadequate treatment facilities sits uneasily alongside a contradictory objection: the pathologization of victims of sexual harms. Sexually abused veterans can only access treatment if they accept a psychiatric diagnosis. In other words, they have to admit that they are mentally ill (*Safety for Survivors*, 2013: 6). Victims reported that military doctors kept asking questions such as ‘how was your childhood’ and ‘Do you have hallucinations?’ (*Safety for Survivors*, 2013: 54). They are told that they are suffering from personality disorders, or are diagnosed with ‘adjustment disorders, bipolar disorder, and many other forms of errant and weaponized psychiatric diagnoses’ (*Safety for Survivors*, 2013: 9). They are branded with the diagnosis of a Traumatic Stress Disorder, even though PTSD-type trauma is only one of the many negative aftermaths of sexual assault: other ailments include depression and anxiety, for example (*Invisible Wounds*, 2013: 20). People lobbying for improved care of victim-survivors constantly maintain that Military Sexual Trauma is ‘not a diagnosis or a mental health condition’ but ‘an experience’ (*Healing the Wounds*, 2010: 19–20), but that message has not permeated military culture.

Victim-survivors object to the fact that treatment consists of ‘nothing but pills and pep talks’: they are ‘fed up with being given endless prescription medication’ (*Healing the Wounds*, 2010: 15). Other victim-survivors resent being sent to mental health clinics. Such clinics are ‘for “crazy” people and they were not crazy’, survivors maintain (*Government Accountability*, 1998: 1). The stigma attached to being sent to psychiatric services is a heavy burden to bear (NAVINSGEN, 2004: vi).

One survivor of sexual assault in the military is Ruth Moore. In 1987, when she was 18 years old, Moore was raped twice by her supervisor. The military response was to diagnose her with borderline personality disorder. She discovered that this ‘was the standard diagnosis that was given to all MST survivors at that time to separate them from active duty and to protect the military from any and all liability’ (*Invisible Wounds*, 2013: 30). Moore’s conclusion was confirmed by Jackie Speier, Representative of California, who also noted that the military ‘find a way to slap them [victims] with a disability of personality disorder, and then discharge them involuntarily’ from service (*Invisible Wounds*, 2013: 44). Servicepersonnel, she laments, understandably ‘do not file complaints because they know what happens’ (*Invisible Wounds*, 2013: 44).

The medicalization of sexual violence focusses largely on the victim, not the perpetrator. This was not always the case. After all, in 1980, PTSD was invented with the aim of turning perpetrators of atrocities in Vietnam into mentally ill patients who were deserving of disability pensions and therapy (see Young, 1997; Bourke, 2007). What is therefore surprising is the relative absence of any discussion in the contemporary period of the perpetrators. Indeed, explanations for why servicemen might act in sexually violent ways are seen as unproblematic: acting in sexually aggressive ways is assumed to be natural, normal, or (at the very most) due to environmental stressors such as poor leadership, male bonding, or unease of being in ‘foreign’ locations.

Finally, the concept of ‘trauma’ as it is applied to victims of sexual abuse does a formidable amount of political and ideological work. One unintended consequence of the rape trauma model is that it places great weigh on social scripts and performativity. The medicalization of victims of sexual abuse requires victims to comport themselves *as* victims. It encourages victim-survivors to perform helplessness, which is not only demeaning but also is in conflict to their ‘warrior’ persona. This means that people who fail to ‘perform trauma’ according to the prescribed script will often fail to have their suffering acknowledged. Victim narratives are accepted so long as they behave *like victims*; the moment they become angry or bitter, they turn into a problem. For female victims, they become ‘feminists’ or man-hating bitches. This was the case with the servicewoman with whom I began this article. Part of the reason she failed to have the harms of being abused recognized was because she responded to trauma by becoming super-effective and composed at work.

Equally, however, the *absence* of a diagnosis of MST or PTSD (especially for victims perceived to be of low status and lacking a powerful, expensive prosecution team) undermines the veracity of their testimony. Typically, it is high-status white, educated victims who can access the psychiatric experts necessary for the effective use of MST or PTSD. Indeed, the fact that some women do not have symptoms of any of these diagnoses has been used as evidence that they were not assaulted. Victims of sexual assault may even demand a trauma diagnosis in order to get counselling, a more sympathetic hearing in a legal review process, or health insurance.

The trauma diagnosis can also open the door to circumventing rape shield laws by allowing investigators to probe into the victim’s sexual or psychological history in attempts to establish another cause of PTSD. The diagnosis can rebound on victim-survivors because it gives the court access to their medical and psychiatric records. It can also open the door to previously traumatic events, such as abortion, to be admitted into court proceedings.

Advocates for sufferers have been understandably frustrated. The most telling critique came from Congresswoman Chellie Pingree in 2013. She wanted sexual assault-induced PTSD sufferers to be treated in the same way as combat-induced PTSD veterans. She reminded Veterans’ Affairs that combat PTSD veterans were only required to provide evidence of three things. They had to be diagnosed as, first, suffering from PTSD and, second, evidence was needed demonstrating a medical link between the diagnosis and the traumatic event. The third requirement was that ‘the claimed stressor must be consistent with the type of events consistent with military service’. With devastating honesty, she contended that ‘the data continues to show that sexual assault in the military is so pervasive that it is consistent with the types of events consistent with military service’ (*Invisible Wounds*, 2013: 51).

*  *  *

There are a large number of factors that cannot be explored in an article of this length. Processes and practices of recognition are shaped by the full range of intersectionalities. This article has emphasized gender, but equally important are race and ethnicity. After all, African American servicewomen are the least likely to have their suffering acknowledged. Rank is crucial: senior women officers are unrapeable and, if their abuse is noticed, is regarded as proof of their flawed leadership style. Unit of service also makes a difference: a trainee at Aberdeen Proving grounds is at greater risk than a female Marine in the field.

This is not to deny that the military has made no advances. In 2005, they established the Sexual Assault Prevention and Response Office (charged with prevention, investigation, accountability, victim assistance, and assessment), introduced mandatory prevention and response training, and improved advocacy for victims. However, these have all been bureaucratic responses which fail to tackle the underlying cultural malaise that permeates military life (González-Prats, 2017: 483–99). The bureaucratic burden is heavy: half of all claims for MST are being improperly processed by the Department of Veterans Affairs; it takes an average of seven years for each claim to be completed; and one in fourteen veterans die while their appeal is pending (Drake & Burgess-Mundwiller, 2019: 661). Most servicepersonnel (of all sexes) remain hostile to initiatives to reduce sexual violence, dismissing them as ‘political correctness’ (Gutmann, 2000). In the words of Maria Caroline González-Prats,

the Department of Defense still maintains that it is the most appropriate institution to fix the issue of MST … despite failing to maintain the revered ‘good order and discipline’ that is claimed to be the cornerstone of its effectiveness as a profession of arms (2017: 495).

Clearly, trauma is a normative concept. It distinguishes ‘normal’ violence from what is excessive and therefore ‘traumatizing’. For example, ‘trauma’ is not assigned to kinds of systemic violence such as in boot camp. It usually requires a sudden event – a violent fissure. Furthermore, it is not only the fact that many underprivileged people have to fight to have their trauma noticed: it is also the case that many people might *themselves* not register an occurrence as distressing simply because it is so typical. Servicewomen have a much higher level of sexual violence *prior* to joining the military: for many, such abuse might seem familiar, even routine. Nightmares, excessive sweating, trembling, flashbacks, and suchlike may be interpreted as part of everyday experiences. In other words, the language of psychological trauma might not make much sense to many people for whom ‘bad events’ are endemic and related to issues such as poverty, patriarchy, and racial prejudice. The idea that there is an ‘after’, a ‘post-’, to abuse is based on an assumption that there is a non-assaultive ‘before’. It assumes that violence is an event limited in time and different from everyday life. Violence of training is seen as ‘necessary’ – for both victims and perpetrators – to create a resilient force.

In the modern military, there are formidable barriers to victim-survivors’ fight for justice. Their suffering is routinely diminished. They are bound to adhere to ‘chains of command’, even when the aggressor may be their ‘next in command’; contractual restrictions mean that leaving abusive contexts is often impossible. Unit cohesion as well as the need to perform tough, ‘warrior’ roles, make any admission of vulnerability dangerous. The medicalization of rape victims *does* make suffering visible, but it does so at the cost of re-inscribing survivors with the stigma of victimhood. This is a particular problem for female victims since it is consistent with the discriminatory military ideology that regards women as lesser beings. They are the vulnerable ‘Other’ which the (male) ‘warrior’ is tasked to protect. On the one hand, victims of sexual assault in the military are regarded as the embodiment of weaker femininity. Obviously, this assumption is strongly resented and resisted by servicewomen, all of whom had chosen (albeit in response to structural vulnerabilities such as poverty and joblessness) to enlist. On the other hand, their identity as female members of a ‘warrior caste’ forces upon them the notion of agency: they are young, active, and strong. The issue of female agency is routinely used in civilian court cases to argue that women are unrapeable: the fit, combat-trained military woman is even more so. They become responsible for their own victimization. All of these prejudices are effortlessly applied to sexually abused male members of the services, too. They are feminized, stigmatized, and cast out of the warrior fraternity. Victimhood becomes tied to the sufferer’s identity – her very selfhood – rather than a description of some harmful event. It adheres to an individual psyche rather than a structural or social failing. As such, it is privatized. The political becomes personal.
